# Differential gene expression patterns in ST-elevation Myocardial Infarction and Non-ST-elevation Myocardial Infarction

**DOI:** 10.1038/s41598-024-54086-w

**Published:** 2024-02-10

**Authors:** Mohammad Elahimanesh, Nafiseh Shokri, Elmira Mahdinia, Payam Mohammadi, Najmeh Parvaz, Mohammad Najafi

**Affiliations:** 1 Clinical Biochemistry Department, Faculty of Medical Sciences, Iran University of Medical Sciences, Tehran, Iran; 2 Cellular and Molecular Research Center, Iran University of Medical Sciences, Tehran, Iran

**Keywords:** STEMI, NSTEMI, Gene profile, Gene network, Systems biology, Biochemical networks

## Abstract

The ST-elevation Myocardial Infarction (STEMI) and Non-ST-elevation Myocardial Infarction (NSTEMI) might occur because of coronary artery stenosis. The gene biomarkers apply to the clinical diagnosis and therapeutic decisions in Myocardial Infarction. The aim of this study was to introduce, enrich and estimate timely the blood gene profiles based on the high-throughput data for the molecular distinction of STEMI and NSTEMI. The text mining data (50 genes) annotated with DisGeNET data (144 genes) were merged with the GEO gene expression data (5 datasets) using R software. Then, the STEMI and NSTEMI networks were primarily created using the STRING server, and improved using the Cytoscape software. The high-score genes were enriched using the KEGG signaling pathways and Gene Ontology (GO). Furthermore, the genes were categorized to determine the NSTEMI and STEMI gene profiles. The time cut-off points were identified statistically by monitoring the gene profiles up to 30 days after Myocardial Infarction (MI). The gene heatmaps were clearly created for the STEMI (high-fold genes 69, low-fold genes 45) and NSTEMI (high-fold genes 68, low-fold genes 36). The STEMI and NSTEMI networks suggested the high-score gene profiles. Furthermore, the gene enrichment suggested the different biological conditions for STEMI and NSTEMI. The time cut-off points for the NSTEMI (4 genes) and STEMI (13 genes) gene profiles were established up to three days after Myocardial Infarction. The study showed the different pathophysiologic conditions for STEMI and NSTEMI. Furthermore, the high-score gene profiles are suggested to measure up to 3 days after MI to distinguish the STEMI and NSTEMI.

## Introduction

Cardiovascular diseases (CVDs) are one of the main causes of morbidity and mortality in the world. Myocardial Infarction (MI), including ST-elevation Myocardial Infarction (STEMI) and Non-ST-elevation Myocardial Infarction (NSTEMI) due to coronary artery stenosis, which is considered the most important factor leading to mortality in CVD patients^1,2^. A vast array of blood factors have been reported as MI general biomarkers in clinical settings^3^. Furthermore, attempts have been made to introduce molecular biomarkers to better distinguish STEMI and NSTEMI.

In the context of patients appearing with signs and symptoms of MI, in addition to clinical examinations, the biological biomarkers help with diagnosis, treatment, and therapeutic decisions^4^. For example, cardiac troponin (cTn) is sensitive for the diagnosis of MI, and is extensively employed in clinical practices^5,6^. C-reactive protein (CRP), as a significant pro-inflammatory mediator, is related to post-MI complications^7^. Creatine kinase MB (CK-MB) is highly present in the myocardium. Following an acute MI, serum total CK level increases after 3 to 6 h. Then, it decreases several days after the onset of MI when there is no further myocardial damage^8^. It is also reported that B type natriuretic peptide (BNP) levels elevate in hypertension, chronic renal failure, and acute MI^9,10^. Many other factors such as the heart fatty acid binding protein (HFABP), Aspartate aminotransferase (AST), Myoglobin, Lactate dehydrogenase (LDH), and Matrix metalloproteinase 9 (MMP9) are suggested as MI biomarkers regardless of distinction between STEMI and NSTEMI^11,12^.

Using high-throughput array techniques might suggest the biological profiles in MI ^13^. The prerequisites for the quick clinical evaluation of serum samples are entirely applicable to the array techniques so that the efforts to find reliable protein and gene profiles have been improved via high-throughput data^14,15^.

Looking at the above studies, there were a set of markers reported in MI (Table 1). The aim of this study was to find the high-score gene profiles and the gene enrichment using the signaling pathways and the Gene Ontology (GO) between STEMI and NSTEMI based on the high-throughput data. Furthermore, it was to introduce and estimate timely the blood gene markers in the STE and NSTE Myocardial Infarction.

**Table 1:** Biological markers suggested by previous studies.

Biomarker	References
Lactate dehydrogenase	^70–74^
AST	^75–79^
CK/CK-MB	^12,80–83^
Troponin	^84–88^
Myoglobin	^89–93^
CRP	^84,94–97^
TNF-a	^98–102^
MPO	^103–107^
MMP9	^11,108,111^
Choline	^112–114^
PAPP-A (Pregnancy-Associated Plasma Protein-A)	^112,115–118^
Plasma neutrophil gelatinase-associated lipocalin (NGAL)	^119–123^
BNP or NT-proBNP	^95,124–127^
Interleukin-6 (IL-6)	^3,128–131^
Soluble CD40 Ligand (sCD40L)	^132–134^
Galectin-3 (Gal-3)	^135–139^
Interleukin-18	^140–144^
Interleukin-37	^114,145–147^
Interleukin-10	^148–152^
Homocysteine	^153–156^
Fibrinogen	^157–161^
F2 isoprostanes	
Adiponectin	^125,162–165^
Apelin	^166–168^
Platelet glycoprotein VI	
Asymmetric dimethylarginine (ADMA) and SDMA	^169–172^
RLPs	^173–175^
Irisin	^176,177^
Lp-PLA2	^178–182^
Cardiac Myosin-Binding Protein C (cMyC)	^183–187^
Heart Fatty Acid Binding Protein (HFABP)	^188–192^
Endothelial cell-specific molecule 1 (ESM-1)	^178,193–195^
Suppression of Tumorigenicity 2 (ST2)	^196–200^
beta-thromboglobulin (beta-TG)	
Cystatin C (cys-C)	^201–205^
Thrombospondin-1(TSP-1)	^206,207^
Syndecan-1( Sdc-1)	^208–210^
LIPCAR	^211,212^
Sirtuin (SIRT1–SIRT7)	^213,214^
Triggering Receptor Expressed on Myeloid Cells (TREML)	^215–217^
Growth-Differentiation Factor-15 (GDF-15)	^183,218–221^
Activin	^177^
PIK3C2A	^222^
PRMT5	^223^
YKL-40	^224–226^
Plasma Mannose	^227^
Glycogen phosphorylase isoenzyme BB	^228^
D-Dimer	^229–233^
Ischemia-modified albumin	^234^
Procalcitonin	^95,235,236^
Secreted frizzled-related protein-5	^237^
miRNA-208 a/b	^238–242^
miRNA-499a-5p	^242–246^
miRNA-1-3P	^239,242,244,247,248^
miRNA-133a/b	^239,246,248–250^
miR-21	^251–255^
miRNA-197	^256^
miRNA-223-3p	^241^
miRNA-328	^249,257^
miR-22-5p	^258^
miR-122-5p	^250,258,259^
miR-19b-3p	^260^
miR-483-5p	^261^
miR-186-5p	^260^
microRNA-224-3p	^262^
microRNA-155-5p	^262^
mir-423	^261,263,264^
mir-223	^265,266^
mir- 186	^265^
mir-150	^267–269^
mir- 486	^268,270^
mir-134	^241,257,260^
miR-92a	^247,271^
CypA (Cyclophilin A)	^272–275^
Mgp (human matrix gla protein)	^276,277^
Ficolin	^278^
Folistatin	^279^
OPG (osteoprotegerin)	^277,280–283^
Pentraxin	^89,278,284–286^
Long non coding RNA (lncRNA)	^287–291^

## Materials and methods

### Text mining data in Myocardial Infarction

**Figure 1:**
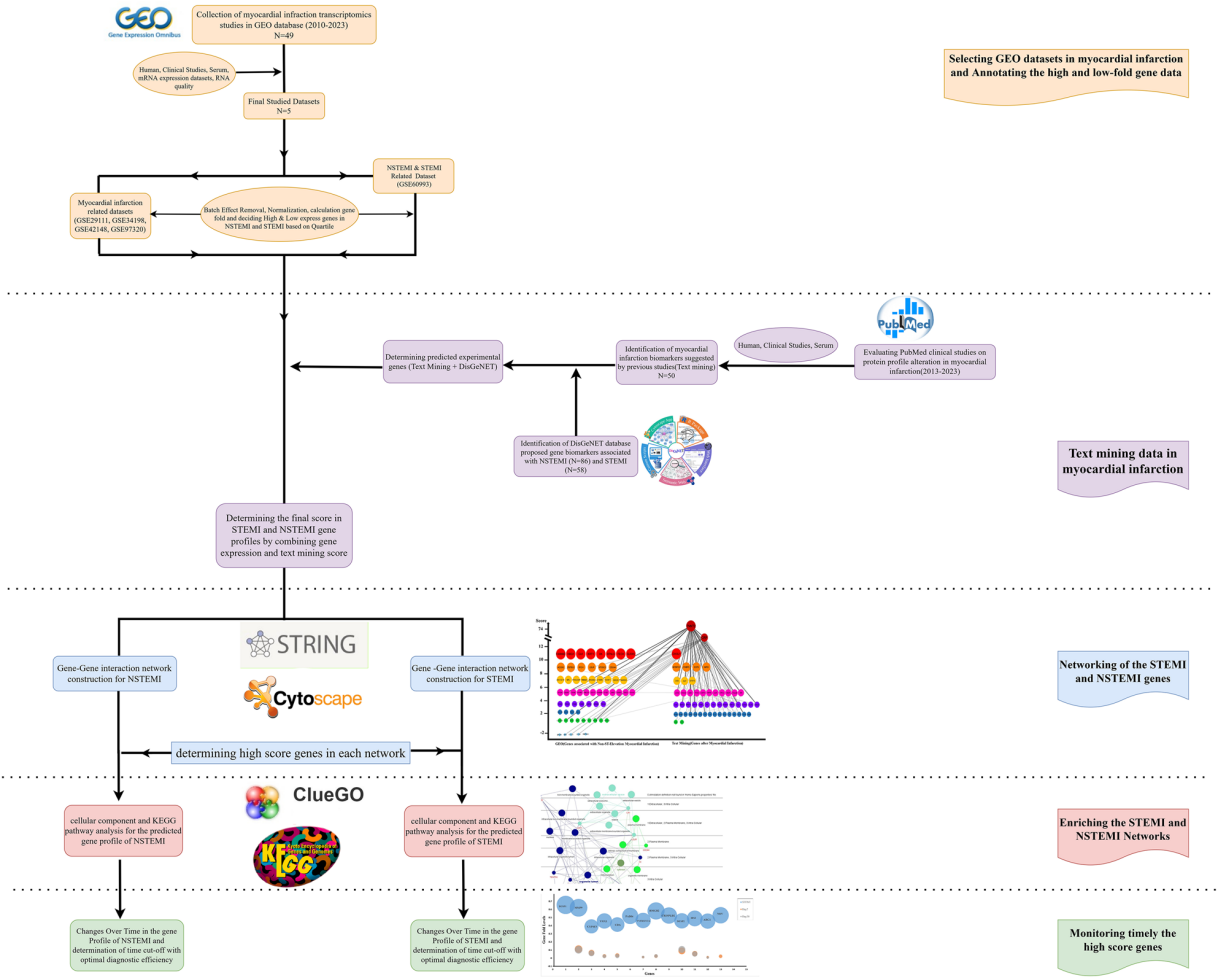
Workflow used for bioinformatics analyses.

The biological genes and compounds released into the bloodstream in MI patients were searched in PubMed between 2013 and 2023. Over 1000 articles were carefully reviewed, and the report frequencies of suggested gene markers were determined during this period. The study followed according to the flowchart in Fig. 1.

#### Merging DisGeNET gene data with the text mining data

The DisGeNET database (https://www.disgenet.org/) is a platform presenting gene data related to diseases based on published clinical trial studies. In this step, DisGeNET gene data of the STEMI and NSTEMI were merged with the text mining gene data. The DisGeNET score relies on the number of clinical trial articles reported to STEMI and NSTEMI (https://www.disgenet.org/biomarkers/). The reports of gene markers obtained from the text mining data were normalized based on the DisGeNET clinical trial reports. Then, the text mining gene score was estimated based on the report frequencies. The DisGeNET gene scores for STEMI and NSTEMI were added to the text mining gene scores, indicating an experiment score.

$$Experiment Score=DisGeNET gene Score+Text mining gene Score$$

### Selecting GEO datasets in Myocardial Infarction

In order to use the transcriptomic data of MI patients, the GEO database was searched between 2010 and 2023. Searching the GEO database was started three years before the search of bibliographic data (text mining) in the PubMed database, since it was proposed that the gene evidence in the GEO datasets might suggest studying the genes in experimental studies. A total of 49 gene expression datasets (including microarray and RNAseq data) were found (Additional File1: S1). Five datasets (GSE60993, GSE29111, GSE42148, GSE97320, GSE34198) were selected (Table 2) based on the following criteria: A) The microarray datasets were obtained from human blood samples after MI. B) The coding transcriptomic datasets were selected. C) The datasets with medical and therapeutic interventions were excluded. D) GSMs (Gene Samples, the datasets in the GEO database include a collection of transcriptomic samples, known as GSMs) with appropriate data quality were selected.

**Table 2:** GEO datasets used in the study.

GEO	Samples	Experiment type	Platform	Year
GSE29111	52	Expression profiling by array	GPL570	2011
GSE60993	33	Expression profiling by array	GPL6884	2015
GSE34198	97	Expression profiling by array	GPL6102	2014
GSE42148	24	Expression profiling by array	GPL13607	2012
GSE97320	6	Expression profiling by array	GPL570	2017

The GSE60993 (GPL6884)^16^ included the blood gene samples of healthy (7 GSMs), STEMI (7 GSMs), and NSTEMI (10 GSMs) subjects, whereas the GSE29111 (GPL570) was associated with the cases 7 days (18 GSMs) and 30 days (18 GSMs) after MI. Two dataset’s raw series were downloaded separately from GEO, and the batch effect was eliminated using the surrogate variable analysis (SVA) package in R Software^17^ (Additional File2: S2, Slides 1 and 2). It is designed to combine data from different datasets, and normalize the gene expression ranges of different samples (https://bioconductor.org/packages/release/bioc/html/sva.html/). The gene fold values for the STEMI, NSTEMI, 7-day, and 30-day (after MI) groups were estimated as compared to the control group according to the following formula (Additional File3: S3).

$${Gene\, Fold}^{1}=\frac{\sum {(GEV}_{Case}-{{\mu }_{GEV}}_{Control})}{{n}_{Case}}-\frac{\sum {(GEV}_{Control}-{{\mu }_{GEV}}_{Control})}{{n}_{Control}}$$

${{\mu }_{GEV}}_{Case}$, *Mean of Gene Expression Values in STEMI, NSTEMI, 7-day and 30-day after MI groups.*

${{\mu }_{GEV}}_{Control}$, *Mean of Gene Expression Values in control group.*

$n$, *Samples in each dataset.*

The high- and low-fold gene levels (> 99.5 and < 0.25 percentiles, respectively) were used to create the gene heatmaps based on the normalized data distribution using the SVA package and calculating the Gene Fold (Additional File2: S2, Slide 3).

#### Enriching the high- and low-fold gene data

The high and low-fold gene data were enriched with the gene fold values of GSE34198, GSE42148, and GSE97320 datasets. There were three control samples and three MI samples in the GSE97320 dataset, 48 control samples and 49 MI samples in the GSE34198 dataset, and 11 control samples and six MI samples in the GSE42148 dataset. After removing the batch effects using R software (Additional File4: S4, Slides 1 and 2), the gene folds (Additional File5: S5) were calculated as described above. Then, the gene average changes in these three datasets were determined and added to the gene Folds^1^ as estimated from the GSE29111 and GSE60993 datasets (Additional File3: S3).

$$Expression\, Score={Gene\, Fold}^{1}+\frac{\sum gene\, fold}{3}$$

### Integrating the text mining and expression gene data

The text mining gene data annotated with the DisGeNET ^18^ were based on the bibliographic reports and, were estimated as the Experiment score. It was added to the Expression score, obtained from gene expression data, indicating Final score.

$$Final\, Score=Experiment\, Score+Expression\, Score$$

### Networking of the STEMI and NSTEMI genes

The primary STEMI and NSTEMI networks were created using active interaction sources (Databases, Experiments, and Text mining) in STRING (version 11.5, http://string-db.org) including high-score (Centile > 25) and low-score (Centile < 25) genes. Then, the STEMI and NSTEMI networks were transferred to Cytoscape software (version 3.9.1)^19^, merged, and reconstructed with edges (Experiment score) and nodes (Final score).

### Enriching the STEMI and NSTEMI networks

The high score expression genes were enriched in both the STEMI (62 genes) and NSTEMI (55 genes) networks by applying the KEGG pathways^20^ in Enrichr (https://maayanlab.cloud/Enrichr/). Furthermore, the high-score gene profiles (NSTEMI, 14 genes; STEMI, 13 genes) were identified in the cellular components using ClueGO (http://www.ici.upmc.fr/cluego) in Cytoscape^21,22^.

### Monitoring timely the high-score gene profiles

Since the time-dependent detection of biological factors differentially marked in clinical diagnostic protocols thus, it is important to monitor the blood gene expression values after Myocardial Infarction (MI). Based on the gene expression data of 7 and 30 days after MI, the changes of high-score gene profiles were evaluated timely in the STEMI and NSTEMI.

### Determination of time cut-off points

The time cut-off points for the gene profiles were estimated at the sigma statistical levels, based on the numbers of standard deviations (sd) from the mean performance of a procedure. It is well known that the total allowable error (TEa) represents the overall permissible errors that might be found in a laboratory result. These include systematic errors (SE) and random errors (RE). The systematic error (SE) is observed due to inaccuracy in equipment calibration while the random error (RE) occurs due to imprecision in the measurement procedure^23^.

$${Total Error}_{Allowable}=SE+RE$$

$$SE=Bias+ \Delta SE$$

$$RE=CV+\Delta RE$$

When the data are normally distributed with a confidence level of 95% and ΔRE = 0, the statistical values of probability of false reject (P_fr_), and Z are estimated 0.05, and 1.96, respectively. Thus;

$${Total Error}_{Allowable}=Bias+(\Delta SE+1.96) CV$$

The CV changes depend on the numbers ($\Delta SE$), indicated as Sigma, when the TE_a_, and Bias are proposed to have constant values. Thus

$$Sigma=\frac{{\text{TE}}-{\text{Bias}}}{{\text{CV}}}$$

Since the STEMI (13 genes) and NSTEMI (14 genes) fold mean values between the 7-day and 30-day gene profiles were not significant, thus the time cut-off points were estimated between the onset of MI and 7 days after MI. On the hypothesis that the fold values of STEMI, and NSTEMI gene profiles might uncertainly change, the CV values were continuously added to the gene fold values based on $\Delta SE$. Then, the data were statistically compared between the gene fold values of the STEMI/7-day and, NSTEMI/7-day by adding each $\Delta SE$ round so that the time cut-off points were found because overlapping not-significantly data.

### Statistical analysis

The statistical analysis was done using R software (version 4.3.0). The SVA package was applied to normalize and remove the batch effects of data. The time-based cut-off points for gene profiles were identified based on the sigma changes and error detection probability (P_ed_)^23^. The changes in gene profiles were established using linear regression and the student’s two-tailed t test. The changes in STEMI and NSTEMI gene profiles were statistically evaluated as significant when the P-value was lower than 0.05. The signaling pathway and GO enrichment analyses were statistically significant when the E-value was lower than 0.05.

## Results

### The biological markers reported for Myocardial Infarction

The blood biological markers (Table 1) reported in MI were identified by searching PubMed. Using the DisGeNET database, four datasets (C1276061, C1561921, C4255010, and C4700045) for NSTEMI and two datasets (C4699152 and C1303258) for STEMI were found. The genes of datasets for each group (STEMI (58 genes) and NSTEMI (86 genes)) were combined and merged only with the blood biological protein markers (50 genes).

### The high- and low-fold gene mapping in STEMI and NSTEMI

**Figure 2:**
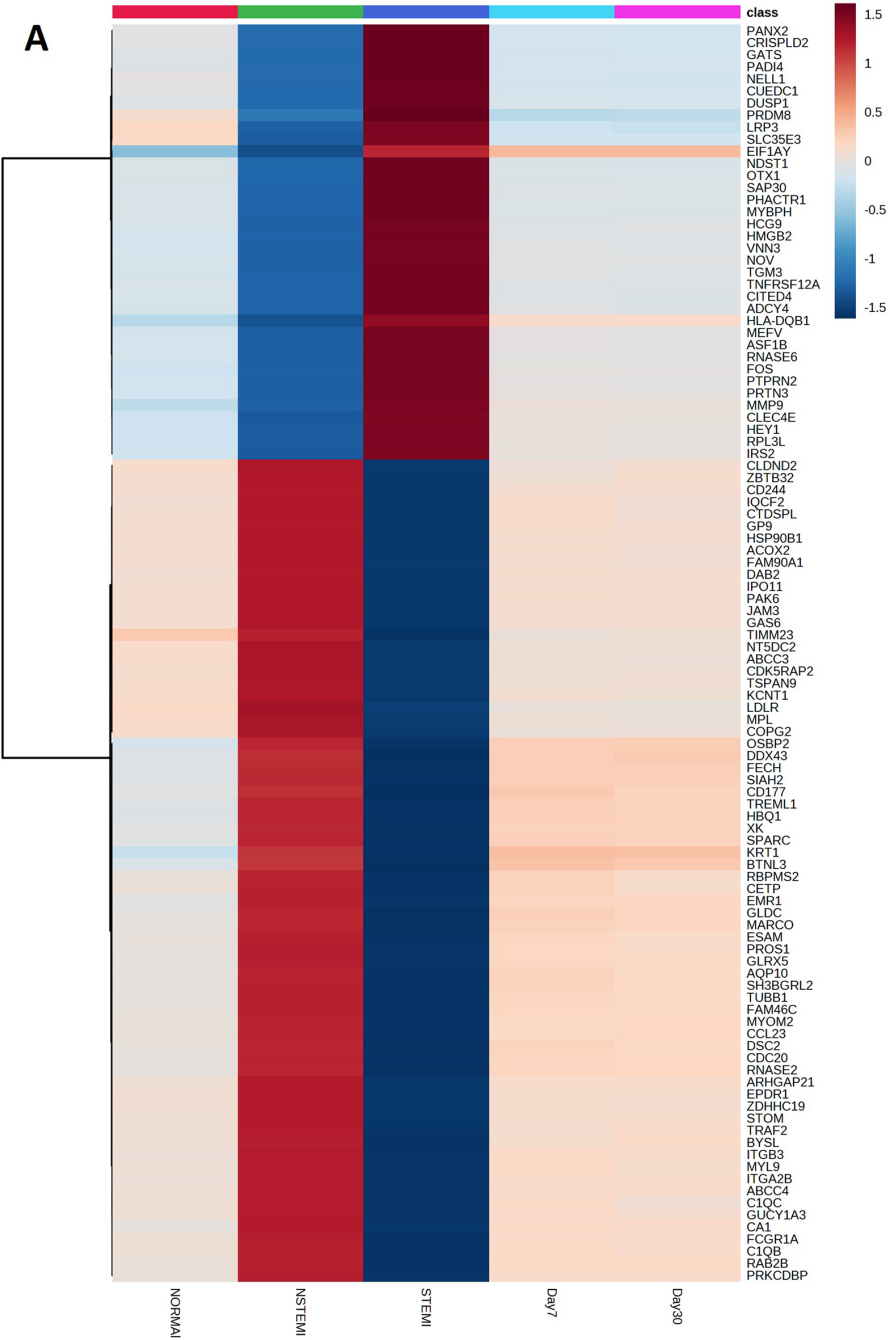

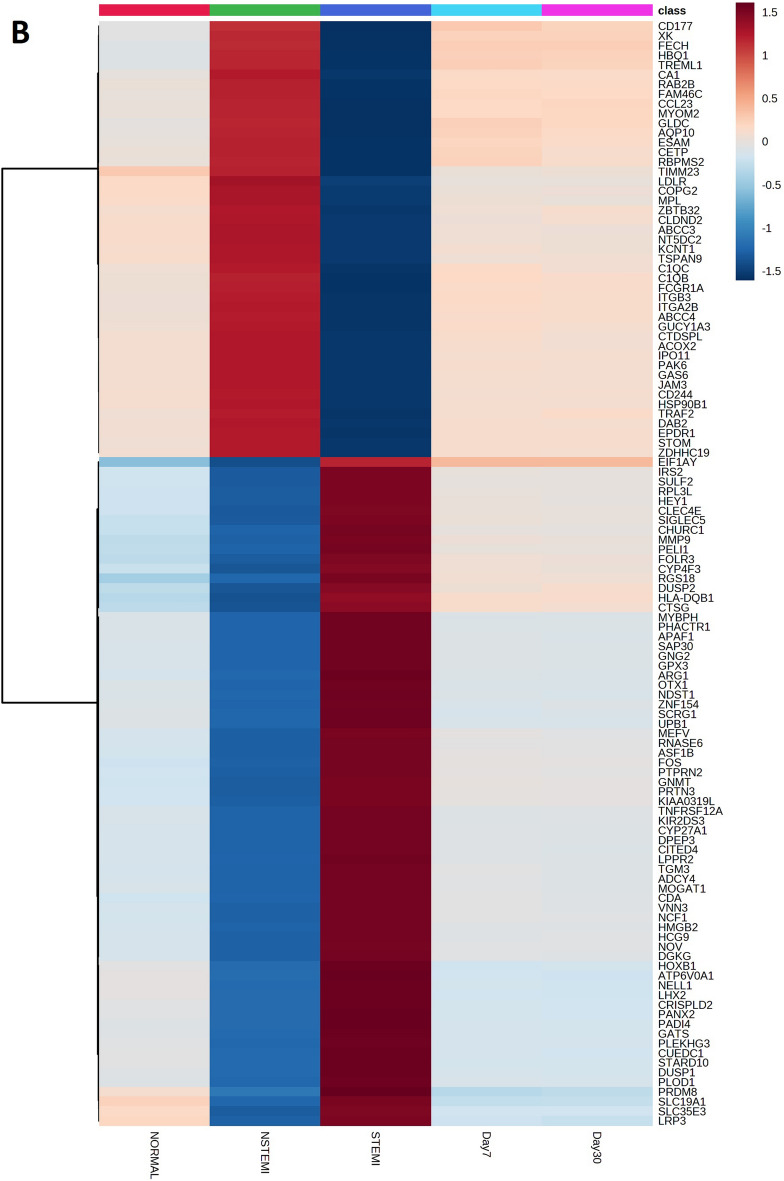
High and low-fold genes in NSTEMI and STEMI. Specifically, the gene folds greater than the 99.5th percentile were assigned as the high fold gene group, while the gene folds lower than the 0.25th percentile were classified as the low fold gene group. (**A**) Heatmap of high and low-fold genes in NSTEMI as compared to STEMI, 7-day and 30-day after Myocardial Infarction. (**B**) Heatmap of high and low-fold genes in STEMI as compared to NSTEMI, 7-day and 30-day after Myocardial Infarction.

The gene expression levels of STEMI, NSTEMI, 7 and 30 days after the MI samples were compared with the control group. The gene heatmaps were presented for the top genes of STEMI (114; high-fold genes 69, low-fold genes 45) and NSTEMI (104; high-fold genes 68, low-fold genes 36) estimated on centiles > 99.5% and < 0.25% (Fig. 2A,B). The STEMI and NSTEMI gene heatmaps revealed clear gene patterns. The gene heatmaps for other groups 7 days and 30 days after MI did not show the differential expression patterns (Additional File6: S6 and Additional File7: S7).

### The gene profiling in STEMI and NSTEMI networks

**Figure 3:**
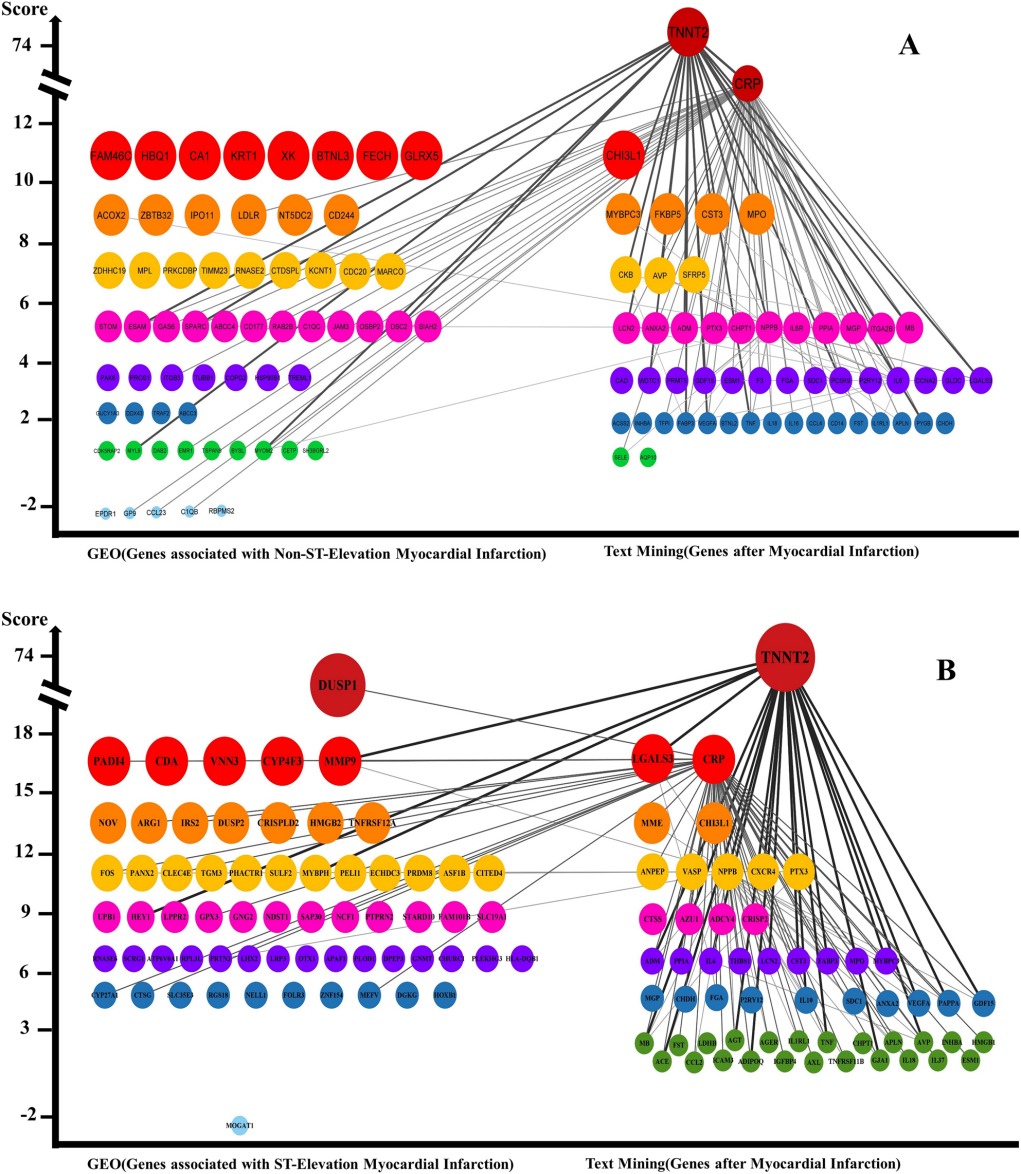
The high-score gene Networks. (**A**) NSTEMI and (**B**) STEMI. The NSTEMI and STEMI networks were constructed by utilizing various data obtained through text mining, DisGeNET, and GEO datasets. The genes on networks were generally divided into two sections. Left, GEO data. Right, the text mining data annotated with DisGeNET database. The Final score represented the node size as indicated on the Y-axis. The thickness of edges reflected the strength of relationships based on the experiment score.

The primary STEMI and NSTEMI gene networks were created using the STRING, and reconstructed on the gene data: (1) The experiment scores of text mining data merged with the DisGeNET (Centile > 25; STEMI, 56 genes and NSTEMI, 53 genes). (2) The low (Centile < 25; STEMI, 1 gene and NSTEMI, 5 genes) and high (Centile > 25; STEMI, 62 genes and NSTEMI, 55 genes) expression scores of the gene data enriched with three GEO datasets. The experiment data (text mining and DisGeNET) showed that the high-score genes in the networks might distinguish STEMI from NSTEMI; however, the numbers of these genes are reported in both STEMI and NSTEMI. Furthermore, the high score gene expression profiles found in the STEMI network (Score > 12; including DUSP1, PADI4, CDA, VNN3, CYP4F3, MMP9, NOV, ARG1, IRS2, DUSP2, CRISPLD2, HMGB2, and TNFRS12A) and NSTEMI network (Score > 8; including FAM46C, HBQ1, CA1, KRT1, XK, BTNL3, FEXH, GLRX5, ACOX2, ZBTB32, IPO11, LDLR, NT5DC2, and CD244). The gene expression data were compared with the experiment data on the networks (Figs. 3A,B).

### Enriching the STEMI and NSTEMI networks with signaling pathways

**Figure 4:**
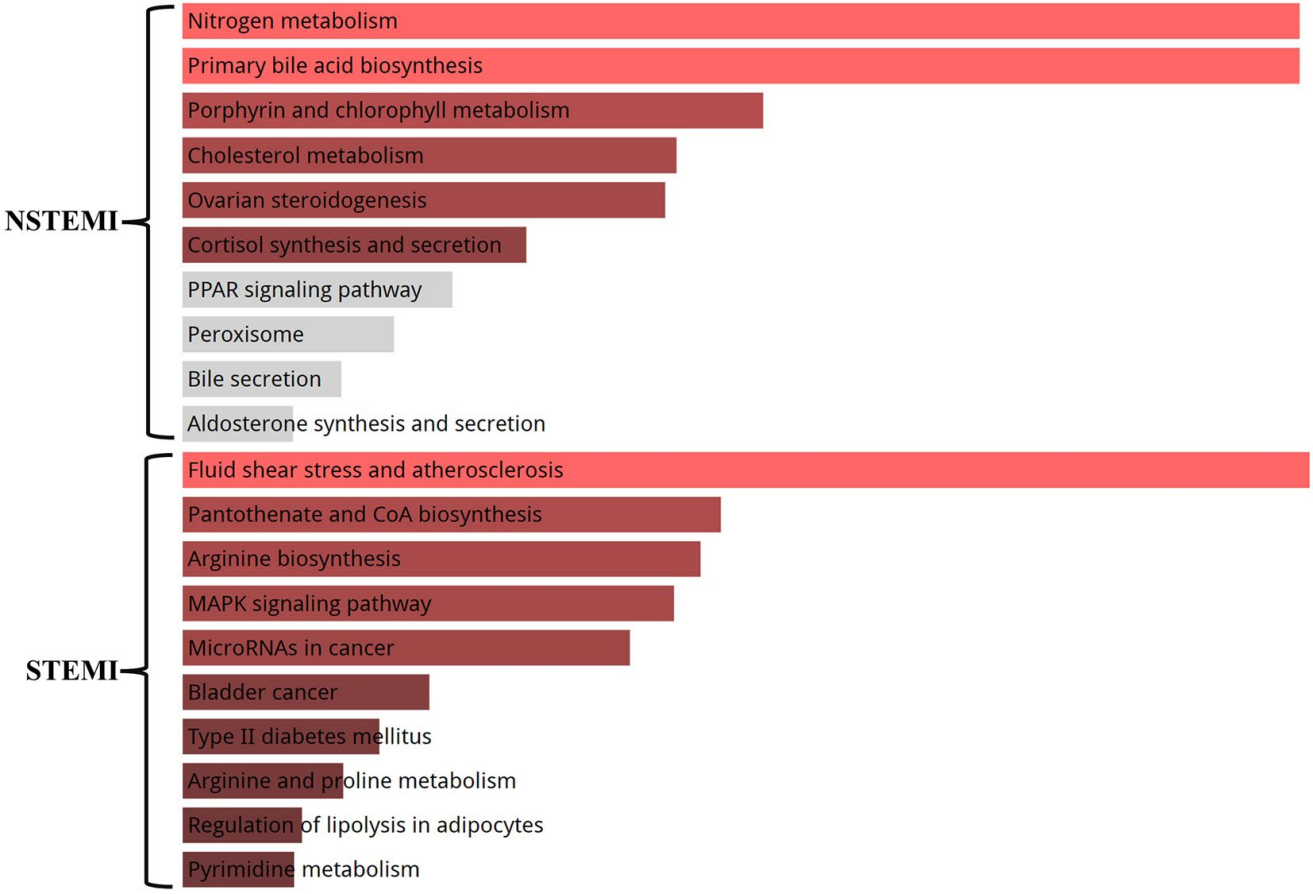
KEGG pathway analysis. Signaling pathway enrichment analysis of the STEMI and NSTEMI high-score genes.

The KEGG pathway enrichment analysis was performed on the high-score gene expression nodes in both the STEMI (62 genes) and NSTEMI (55 genes) networks. The NSTEMI genes were suggested to be associated with certain signaling pathways, namely nitrogen metabolism (E value 0.0254e^−4^), primary bile acid biosynthesis (E value 0.0864e^−3^), porphyrin metabolism (E value 0.0065e^−5^), and cholesterol metabolism (E value 0.074e^−2^). On the other hand, for the STEMI genes, the proposed pathways were fluid shear stress and atherosclerosis (E value 0.0394e^−4^), CoA biosynthesis (E value 0.0012e^−6^), arginine biosynthesis (E value 0.0254e^−4^), and MAPK signaling pathway (E value 0.0011e^−3^). However, the signaling pathway analysis might determine the different cellular functions in ST-elevation and Non-ST-elevation MI (Fig. 4).

### Enriching the STEMI and NSTEMI networks with gene ontology (GO)

**Figure 5:**
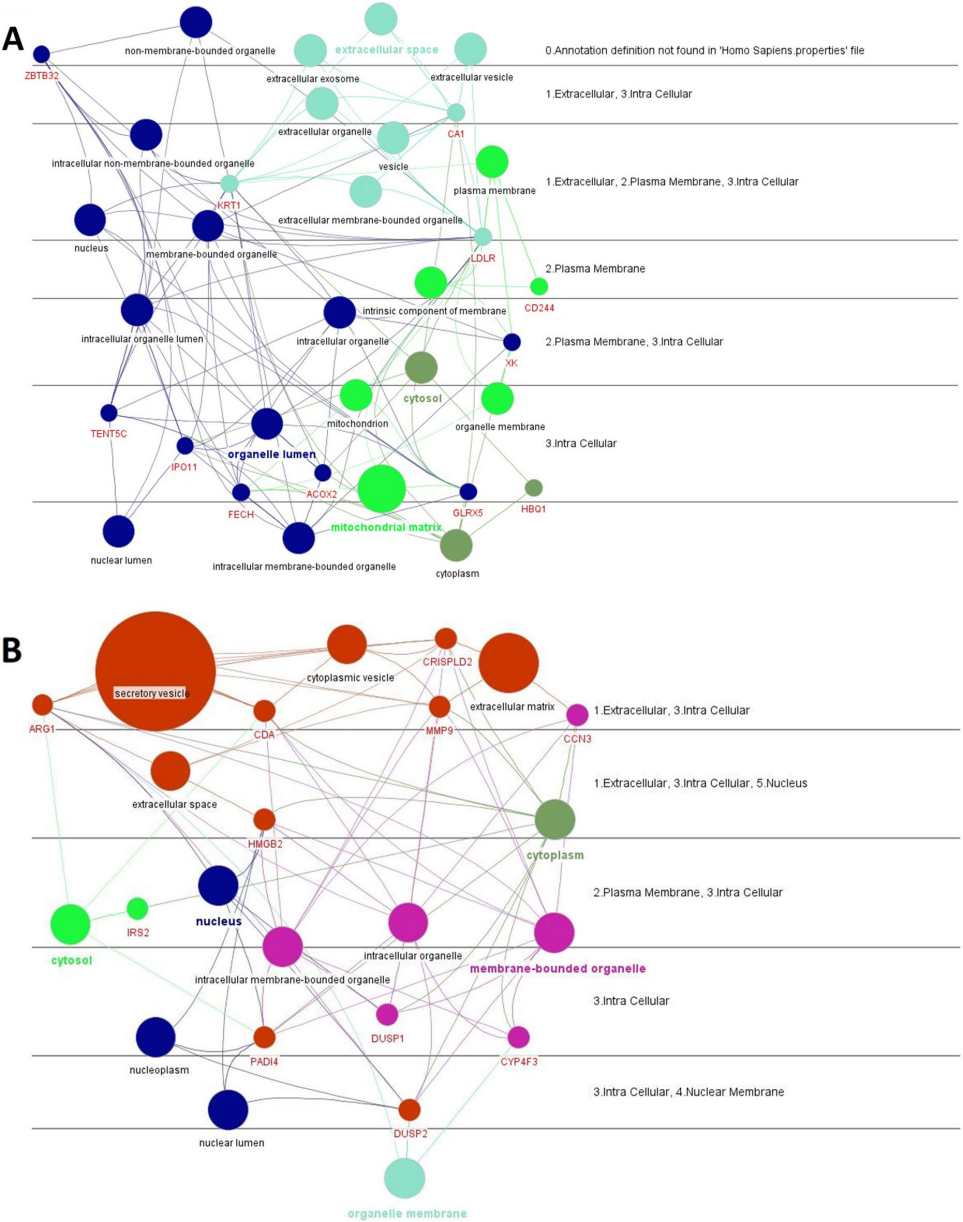
Cellular locations of the STEMI and NSTEMI high-score genes. The localization of (**A**) NSTEMI-associated genes and (**B**) STEMI-associated genes.

The STEMI and NSTEMI high-score gene expression profiles were enriched using GO (cellular component). The NSTEMI genes were found to be more prevalent in several cell compartments, including the mitochondrial matrix (E value 0.02e^−2^), nuclear lumen (E value 0.254e^−3^), cytoplasm (E value 0.0874e^−2^), and intracellular membrane-bound organelles (E value 0.0254e^−3^). On the other hand, the STEMI genes were more abundant in cytoplasmic vesicles (E value 0.524e^−3^), secretory vesicles (E value 0.009e^−3^), and the extracellular matrix (E value 0.134e^−4^) (Fig. 5A,B). The results showed that the frequencies of organelle genes in Non-ST-elevation Myocardial Infarction are more considered as compared to ST-elevation Myocardial Infarction.

### Determination time cut-off points for STEMI and NSTEMI gene profiles

**Figure 6:**
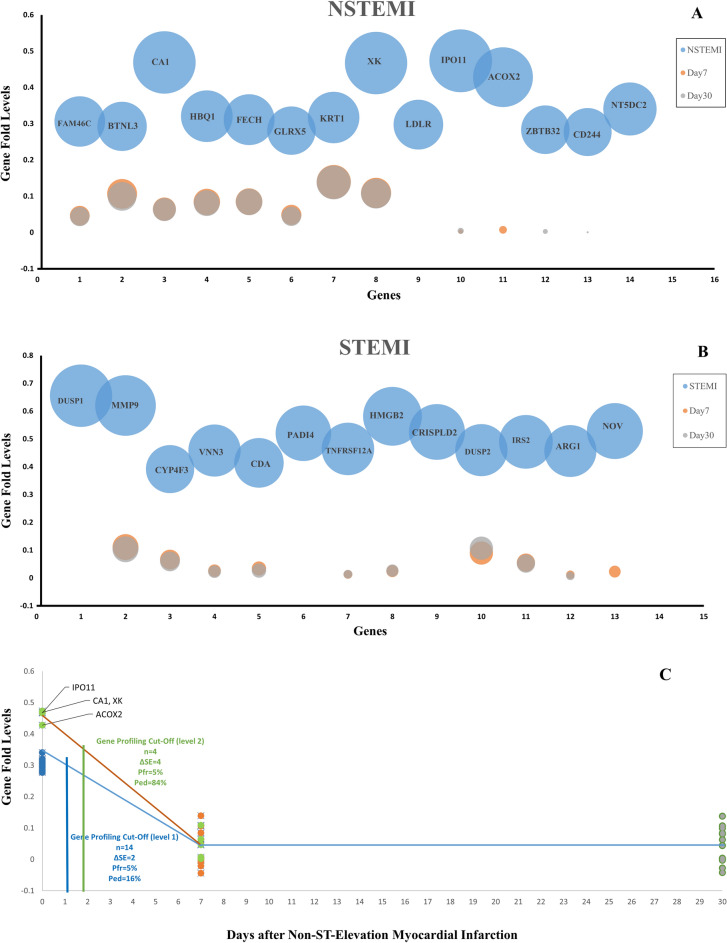

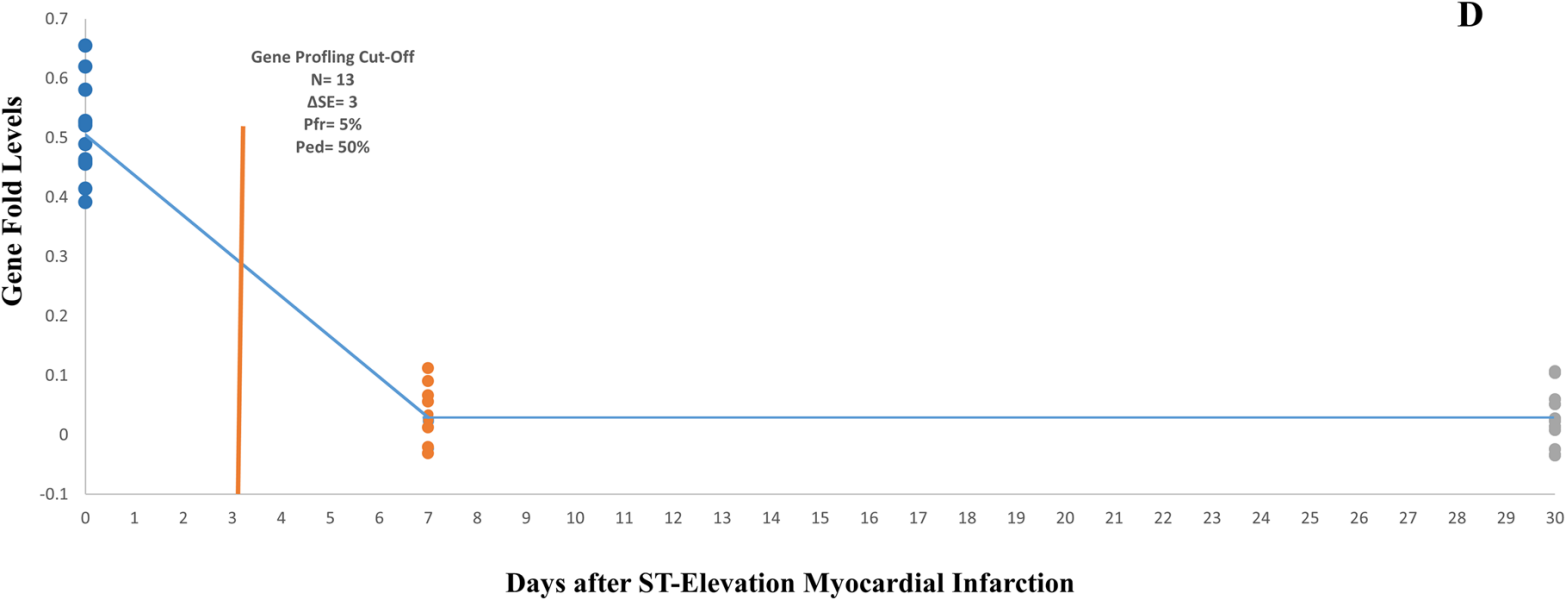
The STEMI and NSTEMI high-fold gene profiles. (**A**) The gene profiles in the NSTEMI, 7 and 30 days after MI. The high-fold genes showed two gene fold levels so that the IPO11, ACOX2, XK, and CA1 genes were in higher fold levels. (**B**) The gene profile in the STEMI, 7 and 30 days after MI. (**C**) The time cut-off points for NSTEMI high-fold gene profiles. According to the gene fold levels, the time cut-off points were 2 days and 1 day for level 1 (4 genes) and level 2 (14 genes), respectively. (**D**) The time cut-off point for STEMI high-fold gene profiles. The time cut-off point was estimated 3 days for the STEMI gene profile (13 genes).

The changes in STEMI and NSTEMI gene profiles were evaluated in three periods: MI, 7 days and 30 days after MI (Fig. 6A,B). The time cut-off points were evaluated for the measurement of STEMI and NSTEMI gene profiles (Fig. 6C,D). The time cut-off points for NSTEMI gene profile (14 genes) were studied at two levels. In the first level, which considered all genes, the optimal performance cut-off point of the gene profile was identified one day after MI (Pfr = 5%, Ped = 16%, ΔSE = 2). In the second level, which focused on four genes (namely IPO11, CA1, XK, and ACOX2) with a higher gene expression fold (> 0.4), the optimal performance cut-off point was almost two days after MI (Pfr = 5%, Ped = 84%, ΔSE = 4). The time cut-off point for the STEMI gene profile (13 genes) was established three days after MI (Pfr = 5%, Ped = 50%, ΔSE = 3).

## Discussion

Myocardial Infarction occurs when the blood flow reduces in the important coronary arteries. It might manifest in the forms of ST elevation Myocardial Infarction (STEMI) and Non-ST-elevation Myocardial Infarction (NSTEMI)^24,25^. Although the diagnosis of MI has risen considerably by applying some specific gene biomarkers such as cardiac Troponin T/I, and CK-MB, but these biomarkers are not specific to distinguish NSTEMI and STEMI. Interestingly, NSTEMI and STEMI have different pathophysiologic conditions, indicating that the development and occurrence of MI may be strongly dependent on the different signaling pathways^26,27^. Therefore, the research on NSTEMI/STEMI-related genes may improve the diagnosis and treatment strategies. Liang et al. reported the differentially expressed genes (DEGs) for STEMI by analyzing two datasets (GSE60993, and GSE61144), and focused on immune cell infiltration^28^. However, these datasets were originally analyzed and recorded by Park et al. in the GEO database^16^. In our study, attempts were made to propose new markers aligned with those presented so that the five datasets were analyzed to report the DEGs in STEMI, NSTEMI, 7 and 30 days after MI. Moreover, the gene expression data were enriched with bibliographic data obtained from text mining and the DisGeNET database to support and suggest the blood high-score gene profiles, and to determine the time cut-off points for the measurement of the gene profiles after MI in STEMI and NSTEMI. Also, the GO and pathway enrichment analyses suggested the cellular pathophysiologic differences between STEMI and NSTEMI.

It is well known that biomarkers are essential in clinical decision making to improve the treatment strategies^29^. Their exceptional accuracy and sensitivity in diagnosing diseases make them highly valuable^30,31^. Some biomarkers of myocardial necrosis that are released into the circulation due to myocyte damage include cardiac-specific troponins T and I, CK-MB, LDH, AST, myoglobin, BNP, Copeptin, Interleukin 6, Interleukin 37, Soluble CD40 Ligand, Heart fatty acid binding protein, protein C binding to cardiac myosin, suppressor of tumorigenesis 2, and cystatin C^31–33^.

According to the study results, the text mining data annotated with the DisGeNET data showed some high-power biomarkers such as troponin, Creatine kinase, CRP, FABP, and myoglobin^27,34–41^. These markers are widely used for MI without the distinction of STEMI and NSTEMI. Furthermore, the text mining data (PubMed and DisGeNET) showed that, however, many genes are seen in both the STEMI and NSTEMI but some genes such as LGALS3, MME, CHI3L3, ANPEP, VASP, NPPB, CXCR4 and PTX3 for the STEMI, and CHI3L1, MYBPC3,FKBP5, CST3, MPO, AVP and SFRP5 genes might be suggested for the diagnosis of NSTEMI. The distinction between these gene groups requires laboratory equipment with high detection limits.

A major blockage in the main coronary arteries causes STEMI, which can lead to heart failure, cardiogenic shock, and sudden cardiac arrest. The danger of death is serious if therapy is delayed, and the blood flow is not immediately restored for the injured portions of the heart muscle. NSTEMI, in contrast, is brought on by a partial occlusion of coronary arteries and is associated with milder heart muscle damage. NSTEMI is a dangerous condition and needs to be treated very quickly in order to prevent further harm to the heart muscle. For this reason, the identification of blood gene profiles is important for the diagnosis, treatment, and management of STEMI and NSTEMI. Some studies reported that the gene expression patterns related to the specific signaling pathways are different in the STEMI and NSTEMI^42–44^. In this study, the fluid shear stress and MAPK signaling pathways were found to be involved in the STEMI gene profile. Previous studies have pointed out that the MAPK pathway activity boosts myocardial ischemia linked to MI^45^. Moreover, it is well known that the endothelial cells relate to the fluid shear stress in healthy blood vessels. A pro-inflammatory response induces abnormal fluid shear stress, such as low or fluctuating shear stress, which can aid in the onset and development of MI^46^. Nitrogen metabolism pathway was also found in the NSTEMI. NO is essential for controlling a variety of blood vessel functions, including thrombosis, inflammation, and vascular tone. It is a crucial molecule in the upkeep of vascular health due to its vasodilatory, anti-inflammatory, and anti-thrombotic functions. However, the proper control of nitrogen metabolism may be essential in the NSTEMI^47^. Metabolic syndrome is a collection of risk factors, including dysglycemia, high blood pressure, high triglyceride levels, and low high-density lipoprotein cholesterol levels, that puts patients at risk for cardiovascular disease. It seems cholesterol, porphyrin, and bile acid metabolic pathways, which can be considered the part of this syndrome, are related to the delayed rational occlusion as reported in NSTEMI^48^.

By analyzing the expression data related to NSTEMI, STEMI, and determining the low- and high-score expression genes in the study, the different gene profiles were suggested for the NSTEMI and STEMI. The study revealed the specific NSTEMI gene profile including FAM46C, HBQ1, CA1, KRT1, XK, BTNL3, FEXH, GLRX5, ACOX2, ZBTB32, IPO11, LDLR, NT5DC2, and CD244 as compared to the text mining data, including TNNT2, CRP, CHI3L1, MYBPC3, FKBP5, CST3, and MPO. The roles some of these genes have been reported in the cardiovascular system^49–55^. Furthermore, the specific STEMI gene profile including DUSP1, PADI4, CDA, VNN3, CYP4F3, MMP9, NOV, ARG1, IRS2, DUSP2, CRISPLD2, HMGB2, and TNFRS12A were comparable to the text mining data including TNNT2, CRP, LGALS3, CHI3L1, and MME. A gene profile including four genes was also suggested as the power one based on the quality control analyses. Some of these genes were reported in the Myocardial Infarction^56–67^.

It is obvious that different signaling pathways are compartmentalized in different cellular organelles. The genes in NSTEMI profile shifted towards the metabolic pathways in intracellular organelles, so many genes were found in the nucleus (ZBTB32, IPO11), mitochondria (CA1, FECH, GLRX5), and cytoplasm (ACOX2). It was proposed that the cellular compensable pathways have enough opportunity to induce the organelle genes in NSTEMI^68^. On the other hand, the sudden discharge in STEMI causes the leakage of cellular transcripts into the bloodstream, which occurs because of the death of heart cells due to a lack of oxygen and nutrients^69^. Accordingly, the genes in STEMI profiles were located in the cellular cytosolic and outer compartments such as the cell membrane (VNN3, TNFRSF12A) and the extracellular matrix (MMP9, CRISPLD2, NOV) with a lower opportunity to induce gene expression.

The results of this study clearly showed that the gene distribution changes timely from onset of MI until 30 days after MI in both the STEMI and NSTEMI. These results are explained by the fact that following MI, the genes originated from heart cells are released into the bloodstream and are gradually removed from it^27^. Identifying the precise time cut-off points for diagnosing MI is a crucial aspect of determining the clinical specificity and sensitivity of biomarkers and the gene profiles. This study estimated the time cut-off points up to 3 days to evaluate the gene profiles in STEMI and NSTEMI.

In conclusion, the study showed clearly the roles of some signaling pathways and their cellular compartments in STEMI and NSTEMI. Furthermore, different high-score gene profiles suggested for distinguishing STEMI and NSTEMI. The time cut-off points for measuring the STEMI and NSTEMI high-score gene profiles were proposed up to 3 days after MI.

## Human and animal rights

No animals/humans were used for this article.

### Supplementary Information

Supplementary Information 1.

Supplementary Information 2.

Supplementary Information 3.

Supplementary Information 4.

Supplementary Information 5.

Supplementary Information 6.

Supplementary Information 7.

## Data Availability

All data are available in the main text or the supplementary materials.

## Abbreviations

MI: Myocardial infarction
CVD: Cardiovascular disease
NSTEMI: Non-ST-elevation Myocardial Infarction
STEMI: ST-elevation Myocardial Infarction
