# Supplementary material for: Differential gene expression patterns in ST-elevation Myocardial Infarction and Non-ST-elevation Myocardial Infarction
Source: Sci Rep. 2024 Feb 10;14:3424. doi: 10.1038/s41598-024-54086-w (PMC10858964; doi:10.1038/s41598-024-54086-w)
Supplement: Supplementary file 2 — Supplementary Information 2. [file 41598_2024_54086_MOESM2_ESM.pptx]

## Slide 1
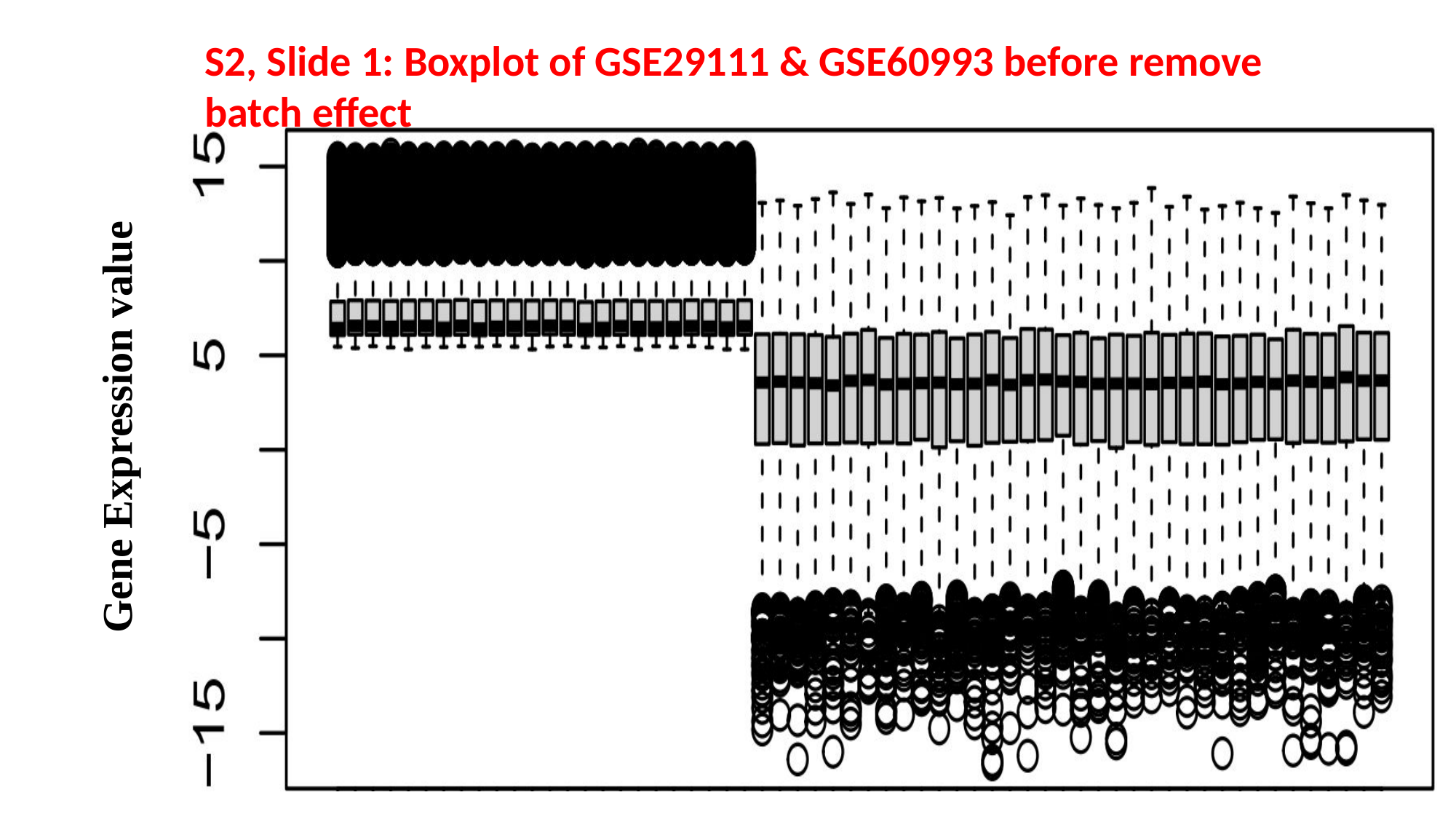

S2, Slide 1: Boxplot of GSE29111 & GSE60993 before remove batch effect
Gene Expression value

## Slide 2
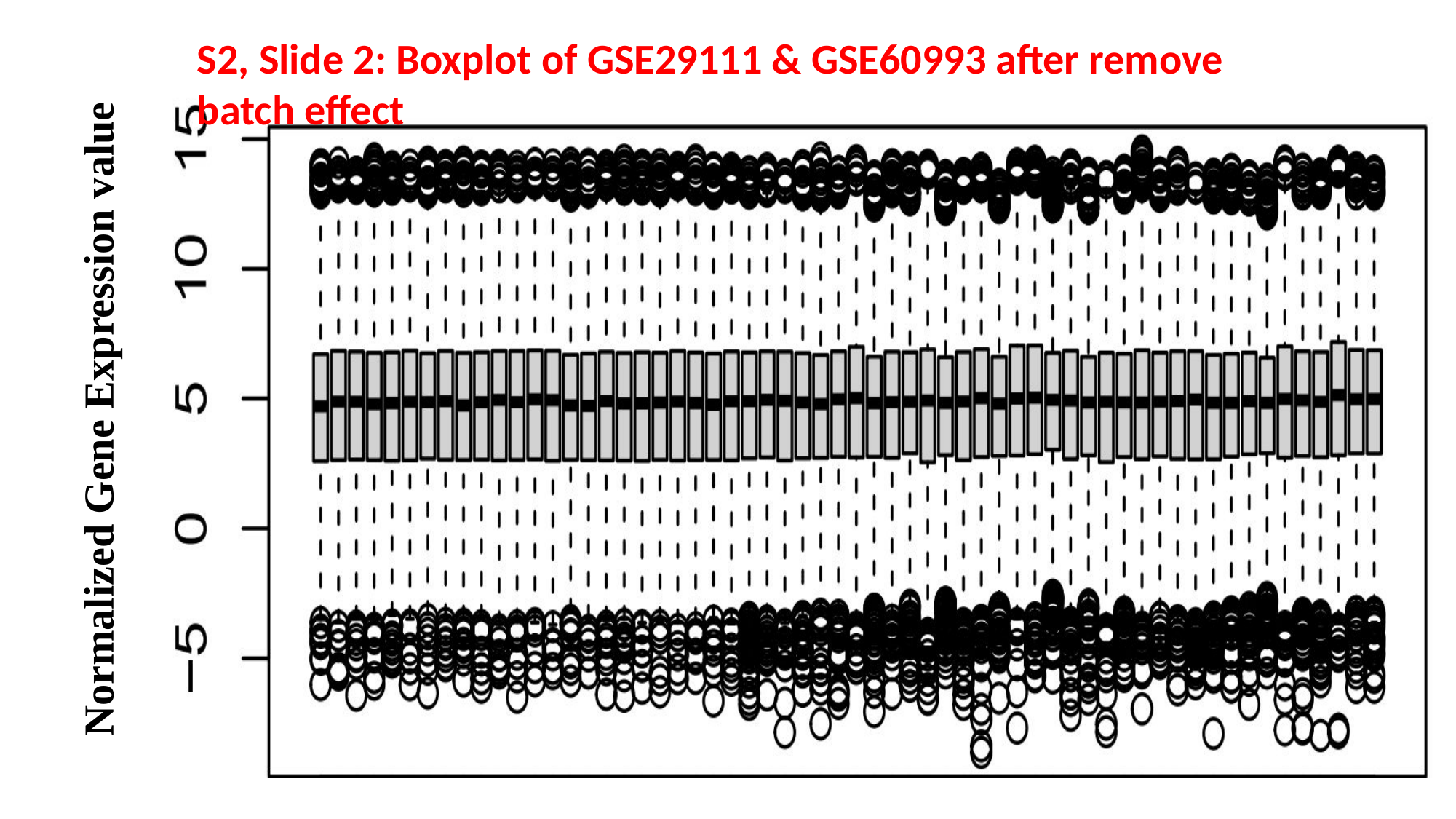

S2, Slide 2: Boxplot of GSE29111 & GSE60993 after remove batch effect
Normalized Gene Expression value

## Slide 3
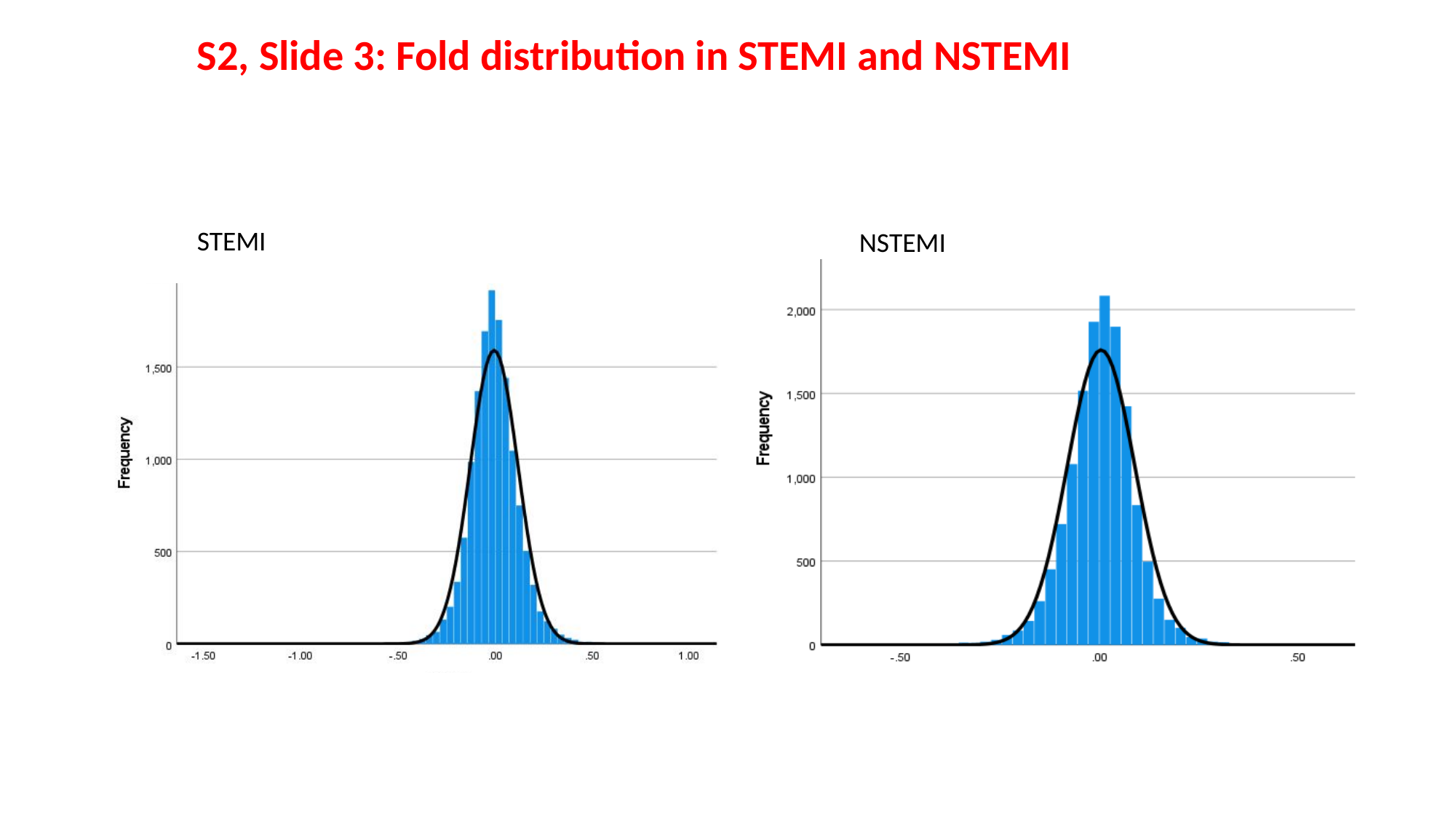

S2, Slide 3: Fold distribution in STEMI and NSTEMI
STEMI
NSTEMI
