# Supplementary material for: Differential gene expression patterns in ST-elevation Myocardial Infarction and Non-ST-elevation Myocardial Infarction
Source: Sci Rep. 2024 Feb 10;14:3424. doi: 10.1038/s41598-024-54086-w (PMC10858964; doi:10.1038/s41598-024-54086-w)
Supplement: Supplementary file 4 — Supplementary Information 4. [file 41598_2024_54086_MOESM4_ESM.pptx]

## Slide 1
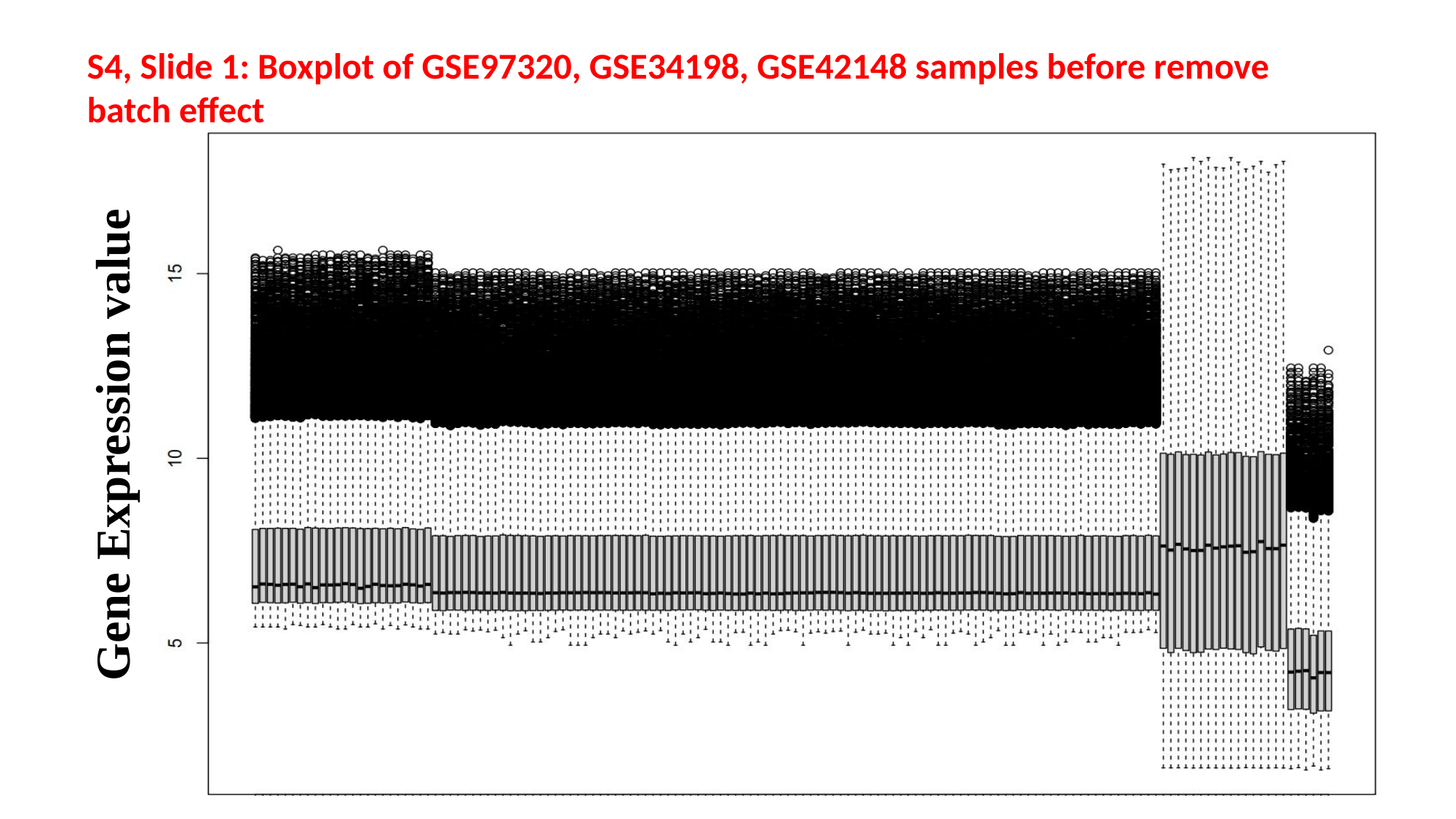

S4, Slide 1: Boxplot of GSE97320, GSE34198, GSE42148 samples before remove batch effect
Gene Expression value

## Slide 2
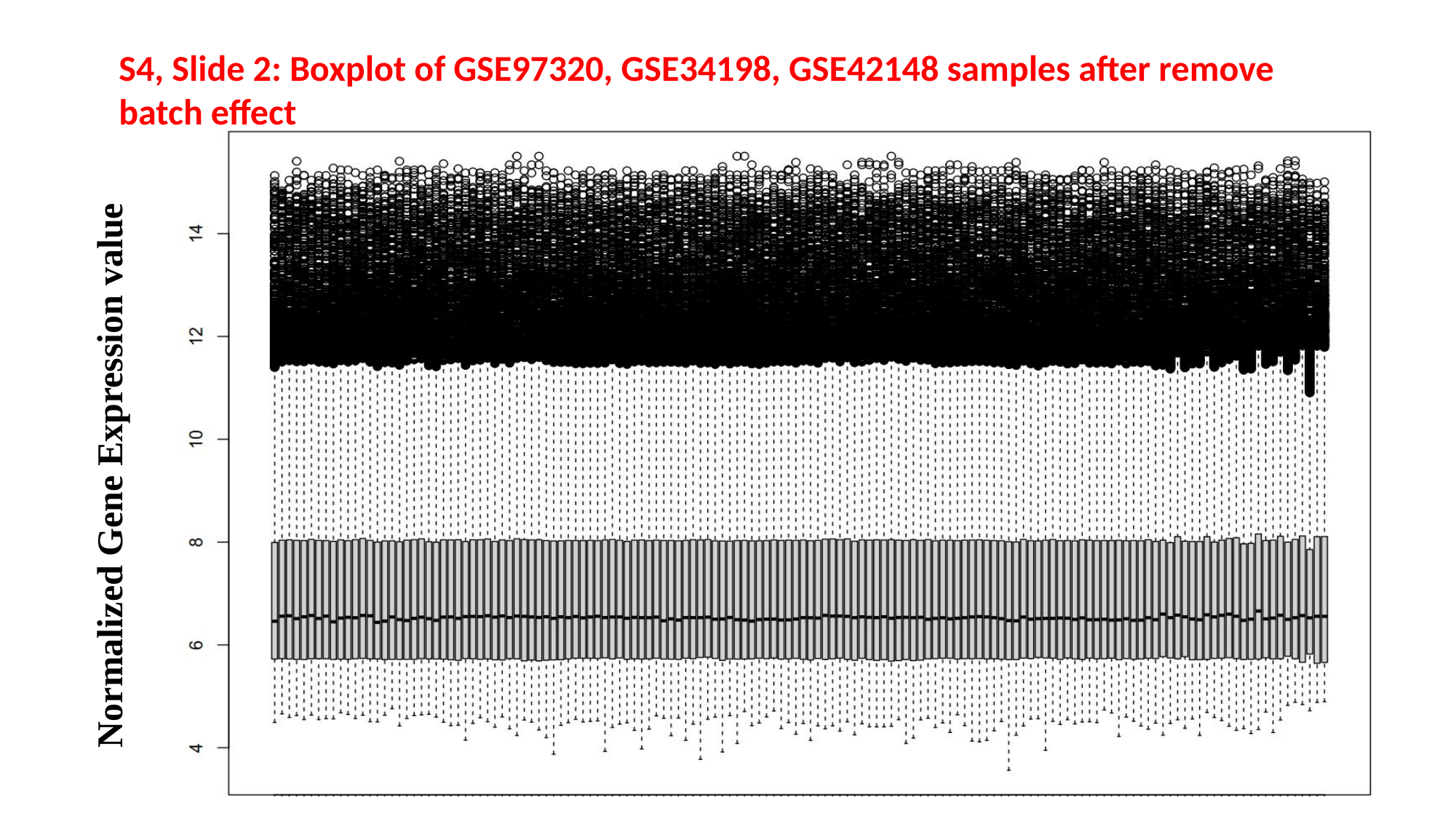

S4, Slide 2: Boxplot of GSE97320, GSE34198, GSE42148 samples after remove batch effect
Normalized Gene Expression value
