# Supplementary figures and images for: Differential gene expression patterns in ST-elevation Myocardial Infarction and Non-ST-elevation Myocardial Infarction
Source: Sci Rep. 2024 Feb 10;14:3424. doi: 10.1038/s41598-024-54086-w (PMC10858964; doi:10.1038/s41598-024-54086-w)

## Slide 1
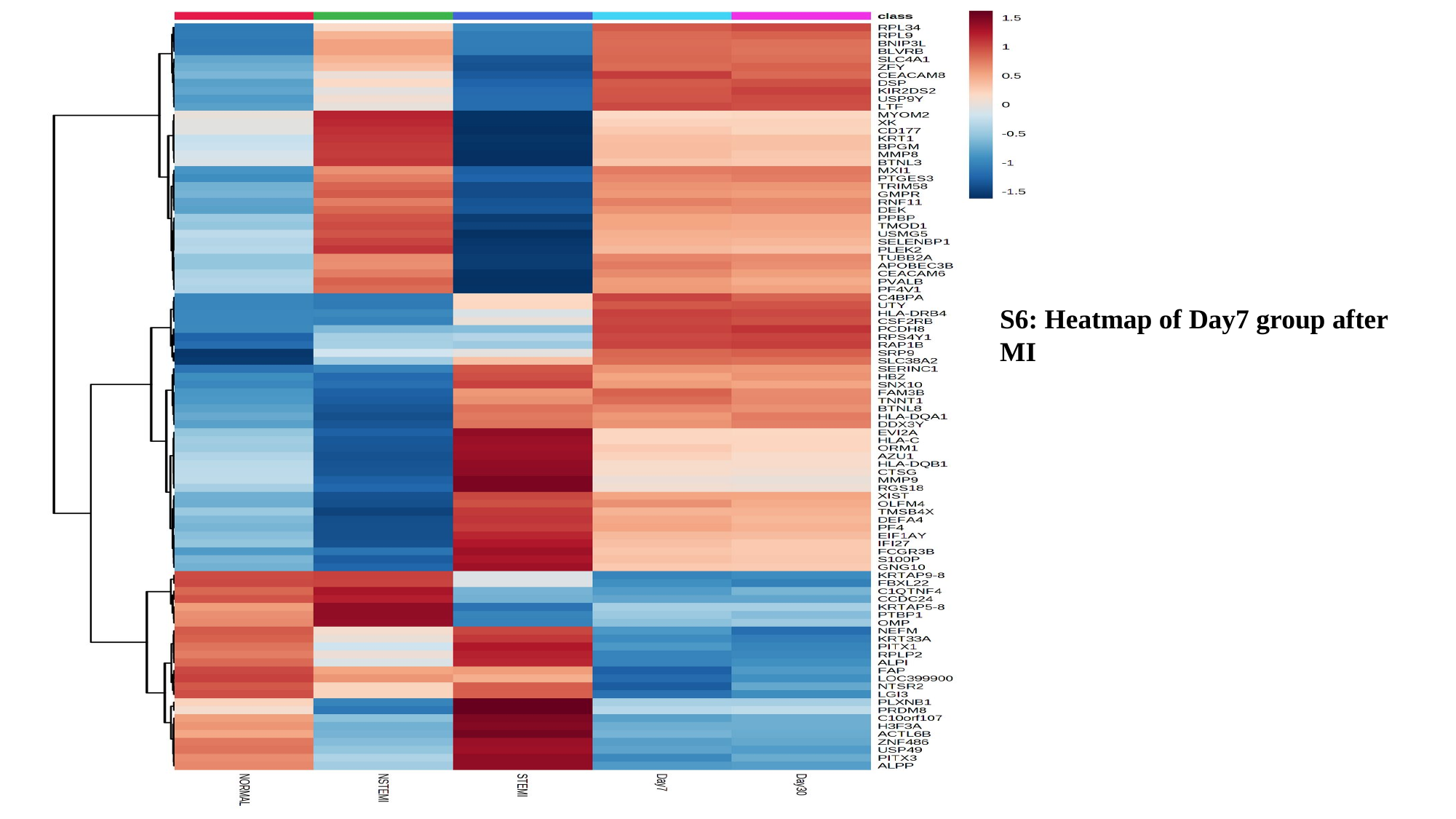

S6: Heatmap of Day7 group after MI

Supplement: Supplementary file 6 — Supplementary Information 6. [file 41598_2024_54086_MOESM6_ESM.pptx]

## Slide 1
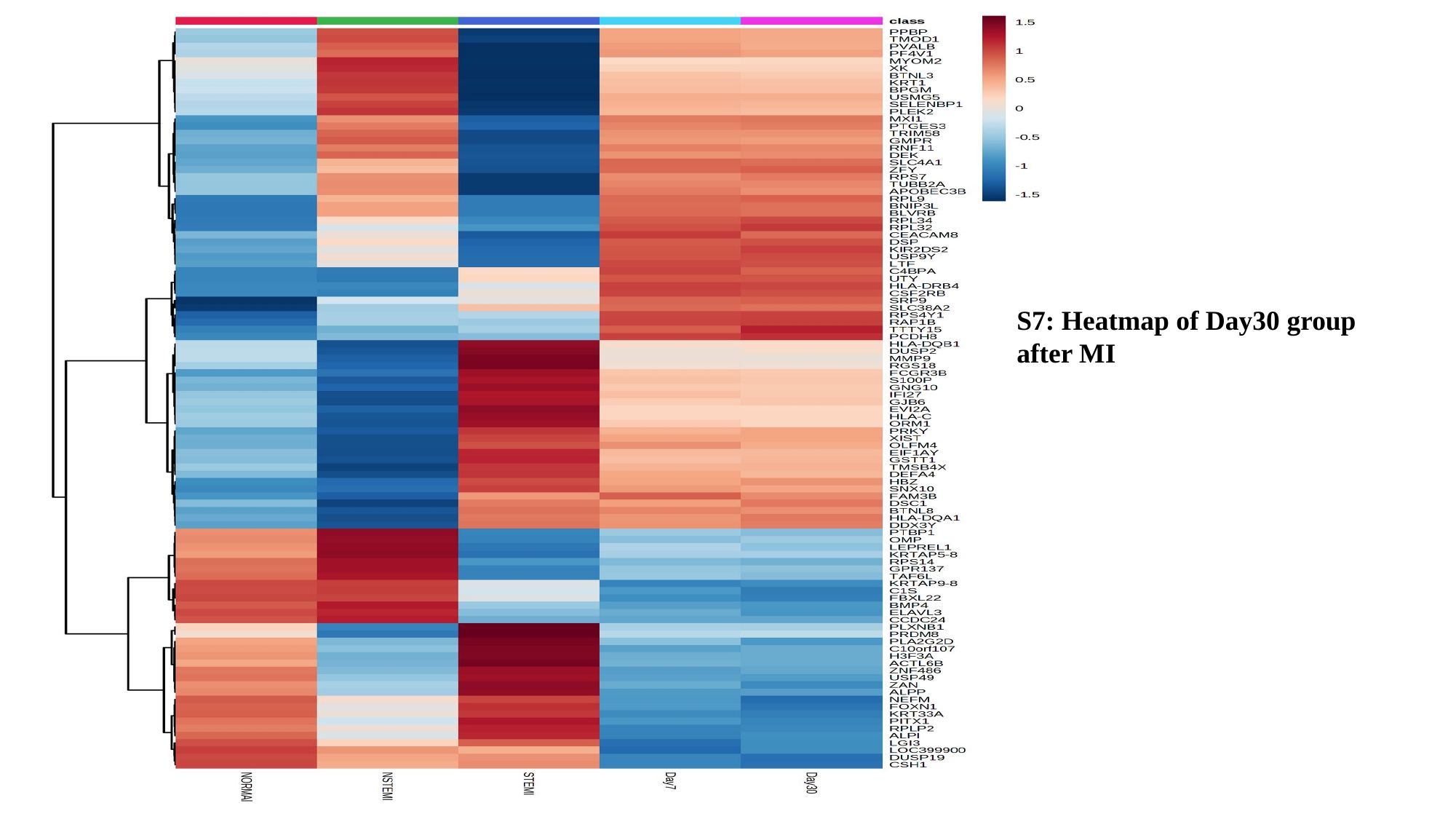

S7: Heatmap of Day30 group after MI

Supplement: Supplementary file 7 — Supplementary Information 7. [file 41598_2024_54086_MOESM7_ESM.pptx]
